# Roles of d-Amino Acids on the Bioactivity of Host Defense Peptides

**DOI:** 10.3390/ijms17071023

**Published:** 2016-06-30

**Authors:** Hao Li, Nuttapat Anuwongcharoen, Aijaz Ahmad Malik, Virapong Prachayasittikul, Jarl E. S. Wikberg, Chanin Nantasenamat

**Affiliations:** 1 Center of Data Mining and Biomedical Informatics, Faculty of Medical Technology, Mahidol University, Bangkok 10700, Thailand; hao8108@yahoo.com (H.L.); nuttapatmt@icloud.com (N.A.); ajaz_me@hotmail.com (A.A.M.); 2 Department of Clinical Microbiology and Applied Technology, Faculty of Medical Technology, Mahidol University, Bangkok 10700, Thailand; virapong.pra@mahidol.ac.th; 3 Department of Pharmaceutical Biosciences, Uppsala University, Uppsala 751 24, Sweden; jarl.wikberg@farmbio.uu.se

**Keywords:** d-amino acid, host defense peptide, antimicrobial peptide, anticancer peptide, diastereomer, HDP, AMP, bioactivity

## Abstract

Host defense peptides (HDPs) are positively-charged and amphipathic components of the innate immune system that have demonstrated great potential to become the next generation of broad spectrum therapeutic agents effective against a vast array of pathogens and tumor. As such, many approaches have been taken to improve the therapeutic efficacy of HDPs. Amongst these methods, the incorporation of d-amino acids (d-AA) is an approach that has demonstrated consistent success in improving HDPs. Although, virtually all HDP review articles briefly mentioned about the role of d-AA, however it is rather surprising that no systematic review specifically dedicated to this topic exists. Given the impact that d-AA incorporation has on HDPs, this review aims to fill that void with a systematic discussion of the impact of d-AA on HDPs.

## 1. Introduction

Host defense peptides (HDPs) have shown great promise as the next generation of broad spectrum therapeutic agents. Interests in this class of immune molecules stems from their effectiveness and versatility. HDPs have been proven to be bioactive against virtually all types of existing pathogens, namely bacteria [1], cancer [2], fungi [3], parasites [4] and viruses [5]. In many cases, the drug resistance exhibited by pathogens was negligibly effective against the activity of HDPs [6,7]. Additionally, evidence has shown that the development of resistance against HDPs is considerably more difficult and slower than that against conventional antibiotics [8,9]. Thus, current results leave little doubt about the great potential of this class of innate immune molecule to form the basis of a novel class of broad spectrum therapeutic agents.

However, several issues must be addressed in order to realize this potential. The shortcomings of HDPs includes low serum lifetime [10], large molecular size (i.e., makes it difficult to reach the target site) [6], potential immunogenicity [11], high production costs [12] and a general need for even better selective toxicity [6]. However, a number of in vivo tests had already demonstrated the high safety and effectiveness of HDP therapy as shown in Figure S1 [13]. Many strategies have been attempted to address the deficiencies of HDPs such as: (i) optimizing the peptide sequence and searching for the minimum length motif via high-throughput screening, computational approach or a hybrid of the two; (ii) conjugating HDPs to homing or internalization motifs; (iii) altering the peptide chain structure (i.e., cyclization and dendrimer formation); (iv) incorporating non-canonical amino acids; (v) modifying the peptide terminals and (vi) using peptide mimics [14,15,16,17,18].

It should be noted that copious reviews exist on the various experimental and in silico strategies for improving the properties of HDPs or d-AA containing peptides in general [6,19,20,21,22,23]. Amongst the improvement strategies, partial or complete replacement of l-amino acids (l-AAs) with their d-amino acid (d-AA) counterpart appears to be a frequently used method that had consistently been shown to improve key parameters of HDP performance, notably selective toxicity and serum stability. In light of the impact of this strategy, it is rather surprising that although virtually all current reviews mentioned the importance of d-AA incorporation, there appears to be no review that is specifically dedicated to the role of d-AAs in HDPs. As such, this review seeks to fill that void by providing a systematic look at the role of d-AAs in the development of HDPs.

## 2. Natural d-Amino Acids (d-AA)-Containing HDPs

Biosynthesized d-AA-containing proteins was first discovered in 1927 [24] and were once thought to be an exception because the presence of stereoisomers contradicts the classical central dogma stating that ribosomes cannot utilize anything else other than canonical l-AAs. However, subsequent research had revealed that not only are d-AAs widely prevalent but also that most organisms are capable of generating diastereomeric proteins [25]. Natural diastereomers can be produced by a variety of mechanisms, for instance, by spontaneous racemization of l-AA residues. This type of diastereomers is primarily located in metabolically inert proteins of aging tissues such as the elastin, myelin and crystallin of elderly people [26] where the original l-enantiomeric proteins had sufficient time for amino acids to form and accumulate [27]. For a long time, this process was thought to be the only manner in which diastereomeric proteins could be created in animals and that the racemization had no functional meaning. However, subsequent research had revealed the importance of D-residues for biological functions.

Before the discovery of diastereomer synthesis in animals, it was known that bacterial cell wall peptidoglycans possessed d-AAs. These form a class of non-ribosomal peptides/proteins where racemase catalyzes peptide epimerization prior to amino acid activation or during chain elongation [28]. Dermorphin from the skin secretions of the tree frog *Phyllomedusa sauvagii* was the first animal discovered to have a ribosomally synthesized diastereomeric peptide [29], which surprised the scientific community at the time because all known evidence indicated that ribosomes cannot utilize d-AAs. Since then, a considerable number of animal ribosomal proteins containing d-AAs were discovered. In all cases, however, the genetic codon encoding the d-AA is the classical one [25]; hence, the genetic material by itself does not provide information about which residue should be a d-AA. Furthermore, there is no evidence that ribosomes are able to directly incorporate d-AAs into a peptide chain; thus, the only way for ribosomally synthesized proteins to actively acquire d-AAs is via modifications of peptides/proteins that contain all l-AAs [30,31,32]. Notably, certain members of the lantibiotic bacteriocin antimicrobial peptide (AMP) family are natural diastereomers that gain their diastereomeric property via post-translational enzymatic modification. Particularly, the AMP nisin belongs to this family and is used as a food preservative [33]. As of now, all available evidences indicated that ribosomal diastereomers are first synthesized as all-l precursors, whereas isomerization occurs through post-translational modification events. Neither direct incorporation of d-AAs into a precursor nor excision of L-residues followed by reinsertion of D-residues has ever been experimentally confirmed [25].

The biological function of natural diastereomers is diverse, where in some cases the function of d-AAs may be unclear while in others (i.e., the stochastic racemization of aging metabolically inert proteins) they may not have any biological significance at all. In the development of synthetic therapeutic HDPs, it is well known that the introduction of d-AAs tends to enhance the activity and stability of the peptide. Similarly, the natural incorporation of D-residues can have the same function. The presence of d-AAs in certain venom peptides of spiders and platypus have been shown to increase both the venom effective lifetime in the prey as well as its potency [34]. The presence of d-AAs can also be crucial for receptor recognition. For example, in the case of dermorphin, the corresponding opioid receptors can bind dermorphin only if the Ala residue at the C-terminal is in the d-form. Synthetic all-l dermorphin does not exhibit any biological activity [35]. Similarly, the presence of d-AAs mediates the receptor-ligand binding of crustacean hyperglycemia hormone CHH [36] and the mouse formyl peptide receptor Fpr that is responsible for vomeronasal pathogen sensing [37].

**Figure 1:**
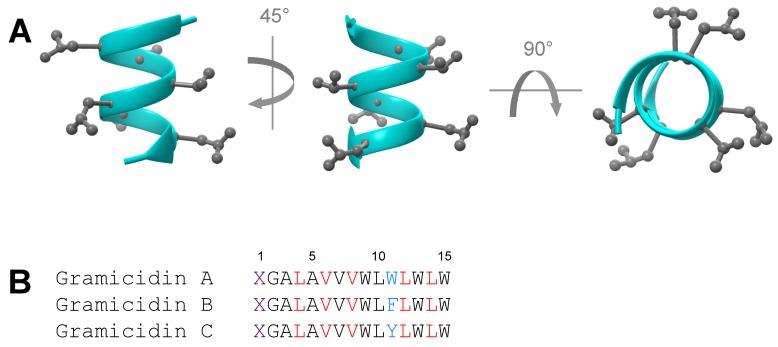
Three-dimensional structure (**A**) and primary sequence (**B**) of Gramicidin D. The first panel shows the protein structure (PDB 1MAG) as a cartoon representation with d-AA shown as grey colored ball-and-sticks. As for the second panel, residues at the first position are represented by purple colored X that can be either valine or isoleucine. Furthermore, l- and d-AA are represented by black and red colored text, respectively, whereas the residue distinguishing the subtype of Gramidicin is indicated by the blue colored text.

Aside from a number of notable exceptions, virtually all d-AA-containing HDPs are synthetic. In particular, to the best of our knowledge, there are no natural HDPs that are composed solely of d-AAs. The family of gramicidin bacteriocin peptides is noteworthy not because it is one of the few naturally occuring d-AA-containing HDPs but because it is the first antibiotic peptide to be used clinically [38]. Gramicidins (Figure 1) are synthesized by the soil bacterium *Bacillus brevis* and are comprised of alternating l-AAs and d-AAs as the general sequence feature. They exert antimicrobial activity via the characteristic HDP mechanism of forming ion channels in cell membranes [39]. Gramicidin D was the first form to be discovered by Rene Dubos in 1939. The D type is in fact a mixture of three linear peptides, namely gramicidin A, B and C. All three possessed d-Leu and d-Val and differ in only the eleventh residue: Trp for type A, Phe for type B and Tyr for type C. Later in 1942, the cyclic S form was discovered by Georgyi Frantsevitch Gause and quickly found application as an antiseptic in the World War 2 [40]. It should also be noted that all gramicidins are synthesized via non-ribosomal pathways. In addition to gramicidin, several other natural diastereomeric AMP types that act via the characteristic HDP mechanism of membrane destabilization also exists such as gratisin GR [41] and the family of tyrocidine [42], both of which are isolated from *Bacillus brevis* (i.e., although gratisin GR originates from the specific strain *Bacillus brevis* Y-33). Furthermore, Bombinin H is isolated from the skin secretion of several species of the *Bombina* frog genus [43]. It is also worthy to note that gratisin GR and all natural tyrocidine subtypes (A, B and C) are cyclic. All natural diastereomeric HDP classes presented here exhibit rather strong hemolytic activity and are therefore unsuited for systemic administration.

## 3. Synthetic d-AA-Containing HDPs

Chemical synthesis gives HDP designers a greater degree of flexibility not found in nature. Virtually any imaginable sequence combination of d-AAs could be envisioned and further subjected to experimental validation. Synthetic d-AA-containing HDPs were found to be effective against the same wide spectrum of pathogens as their all-l counterparts. In many cases, the d-AA-containing HDPs exhibit considerably better selective toxicity than that of their all-l enantiomeric counterpart, as in the case of cancer [44,45], bacteria [1,46], parasites [4] and fungus [3]. The selectivity index of d-AA-containing HDPs is capable of reaching values as high as 400 [9]. Additionally, the serum stability and resistance to proteolytic enzymes had been shown to improve significantly [10,47] upon d-AA incorporation. Furthermore, it can also be effective in addressing potentially toxic immunogenicity, which is one hurdle facing the clinical application of HDPs [11]. Thus, the incorporation of d-AAs into peptides have been shown to improve their pharmacokinetic properties while in some cases may allow peptidess to be administered systemically with great effect [13]. Selected examples of synthetic d-AA-containing HDPs are shown in Table S1. To date, the vast majority of d-AA-containing synthetic HDPs are linear. Particularly, the *α*-helical structure is by far the most common secondary structure found in HDPs, although there are only a few HDPs that have been found so far with secondary structures other than the *α*-helix such as *β*-sheets [9] or *β*-hairpins [48].

## 4. Mechanism of Membrane Destabilization

A number of dedicated studies on the mechanism of membrane destabilization made use of model membranes for determining whether diastereomeric HDPs differ from their all-l enantiomeric counterparts or not. The vast majority of mechanism studies performed so far have been conducted using linear peptides with helical propensity against bacterial targets. This section summarizes and outlines the mechanisms by generalizing the available information from the literature.

At the conceptual level, d-AA-containing HDPs recognize their targets in a manner identical to that of all-l enantiomeric HDPs. The electrostatic attraction of the positively-charged peptide with negatively-charged moieties on the target membrane forms the first step by which the d-AA-containing peptides recognizes and binds the membrane [49,50,51]. Particularly, diastereomers and all-d enantiomers have been tested in this first step against all cellular pathogens, namely bacteria [1], cancer [13], fungi [3] and parasites [4].

For viruses, there is little information on how HDPs recognize and bind to the target. There is an indication that electrostatic binding does have a role in viral recognition. The negatively-charged sialic acids found on a number of viruses were shown to attract HDPs and are possibly responsible for the HDP recognition and binding, which was demonstrated with a scorpion venom peptide recognizing a herpes simplex virus [5]. No information could be found on whether there is a difference in the way diastereomer and all-l HDPs recognize viruses.

**Figure 2:**
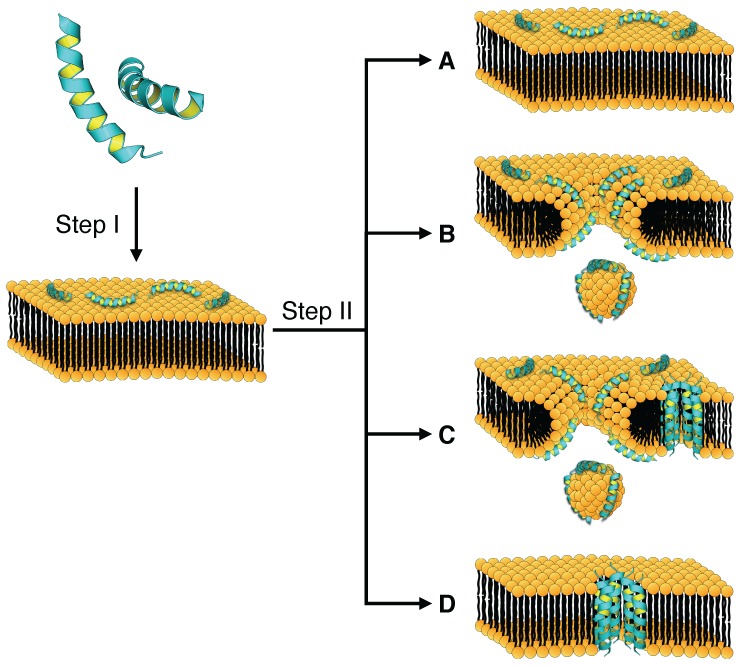
Membrane interaction schematic of diastereomer and all-l peptides with zwitterionic and negatively-charged membranes. Step I: both types of HDP bind to membranes via the electrostatic force, and in the case of all-l peptides, a hydrophobic interaction is also present. Step II: increasing the peptide concentration will result in different outcomes depending on the peptide and membrane type. (**A**) Diastereomer interaction with a zwitterionic phosphatidylcholine and cholesterol (PC/Cho) membrane. The peptide remains at the surface and does not disrupt the membrane structure significantly; (**B**) The carpet mechanism, the primary diastereomer interaction pathway with the negative phosphatidylethanolamine and phosphatidylglycerol (PE/PG) membrane. A large number of the peptides engulf the membrane like a carpet, and their amphipathicity induces micellization. Toroidal pores may also be formed; (**C**) Partial carpet mechanism, the primary interaction mechanism of all-l host defense peptides (HDPs). This mechanism can occur when disrupting both negative and neutral membranes. Both the carpet and membrane pore disruption mechanisms occur. The partial carpet mechanism is also employed by strong hemolytic peptides, such as pardaxin, during the disruption of negative PE/PG membranes; (**D**) Barrel-stave mechanism, the mechanism employed by strong hemolytic HDPs, such as pardaxin, for the disruption of zwitterionic PC/Cho membranes. The peptides insert deep into the hydrophobic core of the membrane and form well-defined trans-membrane ion channel, with the peptide hydrophobic face facing the membrane hydrophobic core and the polar face facing inward. Adapted with permission from [49]. Copyright (2004) American Chemical Society.

Although electrostatic attraction is the overall governing feature in HDP target recognition, there are important subtle differences in how diastereomers bind negatively-charged membranes. Papo and Shai [49] performed a dedicated study with the aim of elucidating the mechanism behind the often profound difference in the selective toxicity of diastereomers compared to that of all-l enantiomer AMPs. The study was performed using model membrane consisting of phosphatidylethanolamine and phosphatidylglycerol (PE/PG) that mimicked a negatively-charged bacterial membrane while the neutral mammalian membrane was represented by phosphatidylcholine and cholesterol (PC/Cho). A schematic representation of the membrane interaction mechanism described in their study is summarized in Figure 2.

Binding analysis measured by surface plasmon resonance on a lipid heteromonolayer and bilayer revealed that l-AA peptides binds similarly to both PE/PG and PC/Cho membranes. However, their corresponding diastereomers binds 50- to 100-folds better to negatively-charged PE/PG membranes than to neutral PC/Cho membranes. Further analysis showed that the all-l peptides binds 10- to 25-folds stronger to zwitterionic PC/Cho bilayers than to monolayers, whereas the diastereomers binds similarly to bilayer and monolayer membranes, indicating that the inner layer of zwitterionic membranes contributes significantly to the binding of the all-l peptides but not of their diastereomers. The findings from Papo and Shai [49] provides a credible, although not necessarily complete explanation as to why diastereomeric AMPs frequently exhibit much better selectivity [44,52] since the lower affinity of the diastereomers toward the neutral mammalian membranes reduces the amount of diastereomers that are bound to healthy mammalian cells and thus less likely to lyse them.

Moreover, l-AA enantiomeric peptides were observed to insert into the PC/Cho bilayers, whereas the diastereomers were surface localized [49,50], thereby providing an explanation on why the inner layer could contribute to the binding of all-l peptides. The outer surface of mammalian membranes is electrostatically neutral; thus, for the l-AA peptide to interact with the inner layer, hydrophobic interactions must dominate once the peptide is bound. Specifically, these interactions are required for the peptide to penetrate into the hydrophobic core of the membrane [22,49]. This finding indicates that the activity of diastereomers depends almost exclusively on electrostatic interactions, whereas the all-l peptides were also capable of a considerable level of hydrophobic interaction for the binding and subsequent insertion into mammalian membranes [49].

Findings from Papo and Shai [49] as well as later works [18,53] had indicated that high hydrophobicity, helicity and amphipathicity were correlated with strong hemolytic activity and cytotoxicity against mammalian cells and therefore exert low selectivity. Furthermore, it should also be noted that diastereomers have lower hydrophobicity and less stable helical structures despite having the same amino acid sequence as their all-l counterparts, which prevents hydrophobic interactions with mammalian membranes and thus imbues the diastereomers with high selectivity. The incorporation of d-AAs thus provides a method of adjusting the parameters that affect selectivity. Additionally, because diastereomers cannot insert into the hydrophobic core of the PC/Cho bilayer, they cannot form transmembrane pores on mammalian cells and must therefore rely on other mechanisms (e.g., carpet mechanism) for membrane destabilization [50].

FTIR analysis of the amphipathicity [16] of the same peptides as studied by Papo and Shai [49] showed that the all-l peptides had an *α*-helical structure once bound to PC/Cho membranes. The diastereomers were also helical but had a distorted $3_{10}$-helical structure that prevented the formation of a well-defined hydrophobic face and hindering its binding to zwitterionic membranes, providing an explanation on why the hydrophobicity of the diastereomers was lower. A similar observation was made by previous works [50,54], which showed that the introduction of amino acids with opposite chirality in the hydrophobic face of amphipathic secondary structures breaks the secondary structures, thus reducing the hydrophobic interaction necessary for interacting with mammalian cells.

The behavior of both diastereomers and all-l peptides were markedly different on negative PE/PG membranes. All-l peptides binds 2.5 to 4 times better to PE/PG bilayers than to monolayers, whereas the diastereomers in this case binds 2 times better to the bilayers as well. Thus, the two peptide types bind to bacterial membranes with similar strength. This finding indicates a weak contribution by the inner layer of negatively-charged membranes to the binding of both peptide types [49] and does provide an indication as to why diastereomers are active against negatively-charged bacterial membranes while sparing neutral mammalian membranes.

## 5. Mechanisms of Bioactivities

### 5.1. Antimicrobial Activity

Once bound to the target membrane, HDPs disrupts the membrane structural integrity and barrier function via a wide range of mechanisms such as pore formation (e.g., barrel stave and toroidal pore) and detergent-like action (e.g., carpet mechanisms), which have already been extensively reviewed [55,56]. Diastereomers and their all-l counterparts have similar membrane destabilization mechanisms. However, the dependence on either membrane perforation or the detergent effect for membrane destabilization is considerably different for the two classes of peptides.

As explained above, diastereomers were found to bind only the surface of simulated mammalian membranes and could not form pores on these membranes. Although the number of peptides tested in the study of Papo and Shai [49] was limited and involved only measurements of model membranes, however it was later shown that the results of this study was indeed general. Circular dichroism analysis performed in a study by Shai and Oren [57] demonstrated that the incorporation of d-AAs was highly disruptive to *α*-helical structures and drastically reduced the helical content of diastereomeric peptides. The absence of significant helical structures prevented the diastereomers from forming transmembrane pores due to the lack of a well-structured hydrophobic face [57,58,59]. Compared to all-l peptides, the diastereomers that were tested on live bacteria had substantially less hemolytic activity, which was notably correlated with the degree of helicity loss, but retained their antimicrobial activity. Scanning electron microscopy confirmed that the membrane lysis was highly significant, thereby proving that the diastereomers had to function via membrane destabilization, despite their inability to form pores. The authors postulated but did not prove that the carpet mechanism was the mechanism of action. The study of Shai and Oren [57] also indicated that the helical structure was important for the cytotoxicity towards mammalian cells but was not a prerequisite for the lytic activity against bacterial membranes. This finding agrees well with the results of Papo and Shai [49].

Similar conclusions were obtained by many related studies [50,51,60,61,62], which had demonstrated via tryptophan quenching that diastereomers rely on the carpet mechanism for membrane destabilization. In this process, a large number of peptide molecules bind and envelope the membrane like a carpet and then dissolves the membrane like a detergent. These studies all demonstrated that the incorporation of d-AAs into peptides destabilizes helical structures. The resulting diastereomers had greatly reduced or even abolished hemolytic and cytotoxic effects against mammalian cells but retained their antimicrobial activity. This result was also applicable to venom peptides, such as melittin [61], which is strongly hemolytic.

Additionally, Oren and Shai [50] suggested that a specific sequence, length, and position of d-AAs were not a prerequisite for antimicrobial activity. Furthermore, a proper combination of hydrophobicity and positive charge may be sufficient for the design of potent antimicrobial diastereomer HDPs. There are, however, some notable contradictions to this view. Chen et al. [14] have shown that the incorporation of a single d-AA into the non-hemolytic HDP magainin could completely inactivate its antimicrobial activity. The loss of activity in this case was accompanied by the loss of the *α*-helical structure. Oren and Shai [50] postulated that this finding may have occurred because magainin already had optimal combination of hydrophobicity and charge; thus, any alteration to the sequence only disrupted the delicate combination and adversely affected its activity.

Oren et al. [51] provided insights into why diastereomers are able to increase the perforation of negatively-charged membranes despite their low tendencies for hydrophobic interactions due to unstable secondary structures (i.e., despite their low tendencies for hydrophobic interactions due to unstable secondary structures) by inserting into the hydrophobic core [49]. Combined Nuclear magnetic resonance (NMR) spectroscopy, Fourier transform infrared (FTIR) spectroscopy, fluorescence quenching and lipid/polydiacetylene colorimetric analyses revealed the following: (i) although the tested diastereomers were nearly devoid of helical structures however diastereomer peptides were able to segregate polar and hydrophobic faces from one another and (ii) helical structures are essential for l-AA peptides to be able to orient their hydrophobic and polar amino acids into separate faces thus allowing hydrophobic interactions with membranes. This separation allowed the diastereomers to insert into the hydrophobic membrane core. The diastereomers in this case were unstructured in solution but were able to adopt a helical structure upon membrane binding. Moreover, their results also indicated that the interaction between the positively-charged diastereomer and the negatively-charged bacterial membranes allowed the peptides to overcome the energy barrier and adopt a stable secondary structure. This finding provides an explanation for the unanswered question of why diastereomers are capable of hydrophobic interaction with bacterial membranes but not mammalian ones [49].

Another effect of the reduced hydrophobicity and amphipathicity resulting from the disruption of secondary structure by d-AAs is the reduction of the peptide aggregation potential in solution [18,63]. Reverse-phase HPLC temperature profiling revealed that high peptide hydrophobicity and amphipathicity is associated with greater peptide self-association in solution, which also correlates with toxicity against mammalian cells [18]. Furthermore, the authors of this study argued that the capacity to decrease peptide self-association in aqueous media also decreases the toxicity against mammalian cells because it allows the peptides to dissociate more easily from each other and associate with the membrane interface, thus preventing further penetration into the hydrophobic core. In contrast, for a negatively-charged bacterial membrane, the interaction of monomeric peptides with the phospholipid head group at the membrane interface was solely sufficient for destabilization. Additionally, no extensive insertion into the membrane core was required such as in the case of the barrel-stave mechanism.

In addition to solution aggregation, Pouny and Shai [63] noted that the capacity to form aggregates inside the membrane was positively correlated with the hemolytic strength of the diastereomers, thereby underscoring the detrimental effect of peptide aggregation on the selective toxicity of the peptide. Furthermore, the results of Chen et al. [18] showed that amongst the different factors (e.g., hydrophobicity and amphipathicity) governing the selectivity of antimicrobial HDPs, helicity had the highest influence. It was found that high helicity was correlated with strong hemolytic activity whereas a reduction in helicity, for instance by the introduction of d-AAs, would correspondingly reduce hemolysis while generally maintaining the antimicrobial activity. It should also be noted that a stable helical structure was not necessary for high selectivity index [57,61,63,64]. The lack of a stable secondary structure and the low amphipathicity of diastereomers suggests that the carpet mechanism and not the barrel-stave mechanism is a requirement for strong and selective antimicrobial activity [18,65].

### 5.2. Anticancer Activity

Aside from the antimicrobial activity, nontrivial aspects on the mechanism of anticancer activity of diastereomeric HDPs have also been explored. A noteworthy mechanism study was carried out by Papo and Shai [66] where they tested anticancer diastereomeric HDPs against small unilamellar vesicles (SUVs) as well as live cells. The SUVs were composed of phosphatidylcholine, sphingomyelin, phosphatidylethanolamine, phosphatidylserine and cholesterol (PC/SM/PE/PS/Cho) lipids and mimicked cancer cell membranes, which possessed approximately 3% phosphatidylserine (PS) content in the outer leaf and thus have a slight negative charge [67]. Utilizing a combination of surface plasmon resonance and confocal microscopy for binding analysis as well as fluorescence spectroscopy and attenuated total reflectance Fourier transform infrared (ATR-FTIR) spectroscopy for structure and membrane permeation analysis, it was found that the diastereomeric HDPs could lyse cancer cells in a manner very similar to that of antimicrobial diastereomeric peptides [66]. Particularly, the peptides first bind the cell membrane via electrostatic interactions (Figure S2) and then dissolved the membrane like a detergent (Figure S3). Additionally, the diastereomers had a distorted helical structure and remained on the surface of the membrane once bound, as also shown in previous studies of antimicrobial diastereomers [51,57].

However, some important differences were found in the anticancer mechanism of diastereomers that may have profound implications for the different design of anticancer HDPs compared with that of antimicrobial ones. In the study, SUVs were loaded with varying amounts of negatively-charged PS ranging from 3% to 20%. A previous study demonstrated that antimicrobial diastereomeric HDPs had up to 100-fold stronger binding to SUVs that mimicked negatively-charged bacterial membranes than to SUVs that mimicked zwitterionic mammalian membranes [49]. Following this logic, one would expect the SUVs loaded with more PS to bind more of the tested anticancer diastereomers. However, the results showed that the diastereomers bind only slightly better (i.e., approximately 2-fold) to the negative model membranes that mimicked cancer cells than to those mimicking normal cells. Moreover, a calcein release assay to detect membrane lysis showed that the tested diastereomers had similar lytic activity on SUVs with 3%–9% PS and that the activity was even lower on vesicles containing 20% PS [66]. These results indicated that the negative charge of cancer cell membranes may not be the sole decisive factor for their recognition. This further challenges the possibility that the leakage of negatively-charged PS to the outer membrane leaflet may be the key element for diastereomer recognition of cancer cells. Particularly, the addition of positive charges at the N-terminus of the peptides did not significantly affect their activity towards live cancer cells. Such procedure is known to increase the antimicrobial activity of diastereomers [66,68,69]. Furthermore, it was found that inversion of the amino acid sequence of diastereomers, which should affect only the dipole moment and thus decrease the helical structure, exerted a profound effect on the anticancer activity. One of the original un-inverted diastereomeric peptides afforded the best discrimination between negatively-charged and neutral SUVs. Nevertheless, this peptide afforded the lowest selectivity toward live cells. In contrast, its corresponding inverted sequence exhibited little to no differentiation between the SUVs, yet it had the highest selectivity for live cells [66]. Thus, the presence of negatively-charged PS in the outer membrane leaflet of cancer cells could not entirely explain the strong selective toxicity exhibited by the anticancer diastereomers as there are more factors involved in the mechanism of action of anticancer diastereomeric HDPs.

The results of Papo and Shai [66] suggested that cancer cell selectivity was correlated with an increase in the level of acidic components such as the *O*-glycosylation of mucins. Nevertheless, the importance of PS in the mediation of the anticancer activity of d-AA-containing HDPs must not be overlooked. Iwasaki et al. [70] tested several all-d enantiomer peptides on several cancer cells whose outer membrane PS densities have been determined using a combination of annexin V-fluorescein isothiocyanate staining staining and flow cytometry. The results showed a very strong correlation between the surface PS density and the oncolytic activity of the peptides thereby indicating that although PS is not likely to be the sole factor in the recognition and lysis of cancer cells, it is an important component of the process. Another interesting finding of this study is that apoptotic chemotherapeutic agents (e.g., dexamethasone) increases the PS content of the outer membrane. This finding hints at a potential method of achieving a synergistic effect by further sensitizing cancer cells to the effect of oncolytic HDPs.

Results from Papo and Shai [66] also showed that the anticancer activity of diastereomer HDPs was not necessarily correlated with the antimicrobial activity because several antimicrobial diastereomers tested in the study were devoid of anticancer activity. Additionally, melittin diastereomers, which had previously been shown to exhibit strong antimicrobial activity [50], were also shown to be inactive against cancer as reported in the study by Papo and Shai [66]. In fact, previous works have shown that unlike the antimicrobial activity, which is primarily affected by peptide hydrophobicity and charge as discussed previously, the anticancer activity of HDPs can be very sensitive to changes in the amino acid sequence [71]. All of these lines of evidences underscore the highly complicated process that are required for recognition of a cancer cell membrane, as such this difficulty must be considered when designing anticancer HDPs de novo.

### 5.3. Antiviral Activity

Membrane destabilization by d-AA-containing HDPs has been observed for all forms of cellular pathogens, namely bacteria [1], cancer [13], fungi [3] and parasites [4]. An exception to this general HDP mechanism of action is of course the mechanism for viruses because this pathogen class does not possess cell membranes. Existing results show that HDPs could exhibit strong inhibitory activity at the viral attachment, entry and notably at the post-entry stages, which is a major challenge in antiviral drug design [5]. Unlike the general mechanism of membrane destabilization against virtually all types of cellular pathogens, the mechanism of antiviral HDPs appear to be less uniform. These HDPs were observed to interfere with the viral reproductive cycle [5], to directly inactive viruses via disruption of viral envelopes (i.e., at least for viruses that have envelopes) [72] and to disrupt viral morphology [5]. The natural diastereomer, gramicidin, has been shown to inactivate HIV at an effective concentration that are three orders of magnitude lower than the cytotoxicity dose [73]. Synthetic antiviral d-AA-containing HDPs (i.e., diastereomer and all-d enantiomer) have also been tested. However, no noteworthy performance difference was observed for the synthetic d-AA analogues in vitro [74,75], which may suggest that the antiviral mechanism is considerably different from the anti-cellular mechanism. The enhanced in vivo performance of the d-analogues could be explained by the increased serum stability rather than the difference in viral inactivation. As of now, there seems to be little if any investigation on the functional mechanism of d-AAs in antiviral HDPs.

### 5.4. Antibiofilm Activity

In the midst of environmental stress, bacteria tend to form multicellular communities known as biofilms in which the living bacterial cells are encased in a protective extracellular matrix consisting of polysaccharides and amyloid fibers. Biofilms render the bacterial cells highly resistant to antibiotics (i.e., up to 1000 folds when compared to the planktonic state [76]) and host immune response as well as being a significant obstacle in the treatment of a large variety of clinical infections [77]. Significant effort is being expended in the search for effective countermeasures and HDPs are amongst the many candidates that are being investigated for the ability to eliminate biofilms [78].

HDPs are capable of disrupting biofilms through multiple mechanisms and at different stages as follows: (i) interfere with the adhesion of the bacteria to surfaces or to other cells in the initial stages of biofilm formation [79]; (ii) directly neutralizing bacterial cells inside the extracellular matrix [79] and (iii) disrupting metabolic pathways related to biofilm formation [80]. It has been suggested by Giovanna et al. [78] that HDPs may have the potential to display all the “ideal” properties of an antibiofilm agent.

As discussed in previous sections, the incorporation of d-AAs in HDPs enhances the therapeutic effectiveness through various mechanisms such as increased resistance to proteases and greater selectivity for pathogen membranes. In the use of d-AA containing HDPs against biofilms, such improvements to the activity is still valid [76]. In fact, a rather large number of studies have reported the use of d-AA incorporating HDPs to disrupt biofilms as a therapeutic route [76,78]. However, instead of directly lysing pathogen cell membranes, the major focus of current antibiofilm HDP research is the disruption of metabolic pathways related to extracellular matrix formation. In fact, a number of potent antibiofilm HDPs (both all-l and d-AA containing) relied on mechanisms other than cell membrane disruption [80,81,82]. Notably, this included a peptide known as 1018, which was capable of completely preventing biofilm formation as well as eliminating established biofilms from both Gram-positive and Gram-negative bacterial pathogens and via synergistic reduction of the antibiotic concentration needed for complete biofilm inhibition by 64 folds [83].

The study by de la Fuente-Núñez [80] presented two synthetic all-d enantiomeric HDPs that were even more effective than the peptide 1018 as well as being found to protect the tested invertebrate animals from lethal *P. aeruginosa* infection in vivo. This study also discussed in detail the mechanism of action of d-AA containing HDPs against the biofilm that was based on the inhibition of stringent intracellular response signal (p)ppGpp. The study observed that direct membrane lytic activity of both all-l and d-AA containing HDPs were not strictly correlated with that of the antibiofilm activity, which is unlike the antimicrobial activity that is directly dependent on membrane lyses. In terms of the underlying physicochemical properties of the peptide, the former is very sensitive to amino acid sequence changes while the latter is reliant on hydrophobicity and positive charge as discussed in the antimicrobial activity section.

### 5.5. Immunological Effects of *d*-AA-Containing HDPs

Although membrane destabilization is the primary pathogen inactivation pathway for HDPs whether as all-l or all-d enantiomers or diastereomers, HDPs also possess other means to defend the host, notably via apoptosis and modulation of a variety of immune responses (e.g., inflammation). The vast majority of research efforts in the development of d-AA-containing HDPs have focused on their membrane lytic activity, but some progress have been made in exploring the potential of their other immune effects. In particular, HDPs can simultaneously possess multiple action pathways. For example, a peptide can simultaneously be an agent for membrane lysis and apoptosis [84,85] or it can simultaneously be a membrane lytic agent and an immune modulator [47,86]. However, there are no report of a peptide that can simultaneously have all three functions.

#### 5.5.1. Immune Modulation

HDPs have been demonstrated to be modulators of a variety of immune responses, such as inflammation and recruitment of dendritic cells, and they are notably able to affect the adaptive immune system by acting as peptide-based vaccine. Human defensins have been shown to stimulate the migration of dendritic cells to papilloma-associated pre-neoplastic epithelium [87] and could elicit an inflammatory leukocyte response, stimulate interferon *γ* production and significantly enhance the antibody response to tumor antigens [88]. Intratumoral injection with a lactoferricin-derived HDP contributed significantly to the infiltration of inflammatory cells into tumor xenografts and subsequently resulted in complete tumor ablation. Mice cured of the initial tumor graft exhibited strong resistance to subsequent tumor re-implantation attempts by the same tumor cells but not by others. Moreover, the resistance was transferable via spleen cell transplantation thereby demonstrating the potential of HDPs as vaccines [86].

Although few studies have been carried out, d-AA-containing HDPs, both as all-d enantiomer and as diastereomers, have also been shown to be capable of modulating immune responses. Lee et al. [47] demonstrated the antiinflammatory effect of an all-d protaetiamycine analogue. The d-enantiomer possessed potent selective bacterial lytic activity and could inhibit nitrogen oxide production (NO) and lipopolysaccharide (LPS)-stimulated macrophage-mediated inflammation and cytokine production. Interestingly, although the direct antimicrobial activity was nearly the same for the d- and l-enantiomers, the antiinflammatory activity of the d-enantiomer was considerably higher than that of the l-enantiomer (Figure S4). Fluorescence analysis revealed that when compared to the l-enantiomer, the d-enantiomer had stronger binding to LPS, a finding that correlates well with the d-enantiomer antiinflammatory activity.

A similar antiinflammatory effect has been observed by Wang et al. [52] when testing a series of diastereomer AMPs. The diastereomers effectively inhibited LPS activation of macrophages, NO production and inducible NO synthase (iNOS) mRNA expression while at the same time maintaining selective bacterial lysis activity. Increases in the hydrophobicity and *α*-helicity correlated positively with the antiinflammatory activity but not with the bacterial lytic activity. This observation hints at the challenges facing the design of multi-functional HDPs because previous studies had established that an increase in hydrophobicity and helicity was detrimental to the selective membrane disruption of HDPs [49,53]. An interesting side note from the study of Wang et al. [52] is that when testing the resistance of diastereomers against proteolytic enzymes, the authors found that a minimum d-AA content of 33% was required to provide the diastereomers with complete immunity against enzyme digestion.

As of now, there seems to be no report of immune cell migration activation or vaccine-like properties of d-AA-containing HDPs in animals, neither as an enantiomer nor as a diastereomer. Yang et al. [89] tested a number of temporin-related peptides and found that the tested peptides were unable to induce the chemotactic migration of human neutrophils and monocytes when the peptide sequence was reversed or when d-AAs were incorporated into the peptides to generate the all-d enantiomers. This finding indicates receptor-dependent activation of this type of immune response that is chirally dependent.

#### 5.5.2. Apoptotic Activity

HDPs are capable of inducing apoptosis in neoplasm cells (Figure S5). For example, lactoferricin B has been demonstrated to induce apoptosis in human cancer cells by depolarization of the mitochondrial membrane [90]. The membrane lysis has been proven to occur independently of the apoptotic effects because treatment of cancer cells with anti-apoptotic agents did not inhibit the peptide activity [85]. One reminder here is that this review discusses the apoptotic effect of HDPs, peptides that bear the hallmarks (i.e., positive charge and amphipathicity) and thus are capable of neutralizing pathogens via cell membrane depolarization. It should be noted that other types of apoptotic peptides were not considered.

d-AA-containing HDPs are also able to induce apoptosis in cancer cells. Kim et al. [91] tested an all-d analogue of the insect defensin, coprisin, and observed chromosomal DNA fragmentation and the expression of transcripts of inflammatory cytokines (e.g., TNF-*α* and IL-1*β*) in human leukemia cells. Simultaneously, the d-coprisin analogue maintained antimicrobial activity against a range of multi-drug-resistant bacteria. However, the study provided no insight into how the d-coprisin entered the cancer cells to initiate the apoptosis. Compared to the l-form, the d-analogue provided considerably higher selectivity in its direct antimicrobial lysis activity.

Aside from causing apoptosis in cancer cells, the all-d analogue of the AMP, bovine myeloid antimicrobial peptide (BMAP-28), was able to induce apoptosis in the protozoan parasite *Leishmania* in both the free-living promastigote and the intracellular amastigote form [4]. Mouse macrophages infected with *Leishmania* treated with BMAP-28 had up to 68% reduced amastigote burden. The d-form of BMAP-28 was more effective against both promastigotes and amastigotes of *Leishmania*. BMAP-28 induces apoptosis in *Leishmania* in a caspase-independent manner. Initially, parasite membrane disruption is observed, with a significant sign of apoptosis as detected by a terminal deoxynucleotidyl transferase dUTP nick end labeling (TUNEL) assay only 24 h after the initial peptide exposure. This finding indicated that the peptide also relied to a significant extent on direct parasite lysis. However, this study also did not discuss how the peptide entered the cytoplasm of the pathogen to initiate apoptosis.

As of now, all-d HDPs have been shown to induce apoptosis, but no report has been found for an apoptotic diastereomer. However, it is very likely that a diastereomer HDP will be able to induce mitochondria-dependent apoptosis should it cross the cell membrane because the membrane structure of mitochondria is quite similar to that of the prokaryotes from which they are believed to have been evolved from [92] and because mitochondrial disruption can result in the release of apoptotic factors that are normally sequestered within the mitochondria [93,94]. An interesting way to provide AMPs with the capacity to disrupt mitochondrial function is by conjugating them to an internalization motif. Chen et al. [95] conjugated the horseshoe crab AMP, tachyplesin, to the integrin homing domain Arg-Gly-Asp (RGD). The resulting construct was effective at inducing apoptosis in tumor cells. JC-1 staining confirmed the disruption of the mitochondrial membrane, and Western blotting revealed the presence of caspase and Fas apoptotic factors.

Thus in summary, in addition to their strong, selective membrane disruption activities, d-AA-containing HDPs possess notable immune modulation activity and are capable apoptotic agents. A thorough elucidation of the mechanisms responsible for these activities remains to be carried out.

### 5.6. Influence of Secondary Structure on Bioactivity

Currently, the vast majority of studies dedicated to the mechanism of d-AA incorporation into HDPs had been performed on linear peptides with helical propensities and on peptides targeting either bacteria or, to a limited extent, cancer. Huang et al. [96] had investigated the role of helicity and hydrophobicity on the bioactivity of anticancer peptides by showing that d-AA can be used to improve peptide specificity toward cancer cells, particularly by substituting them on the polar face of the helix to modulate their cytotoxicity while its placement on the non-polar face could still maintain the anticancer activity. Similarly, Huang et al. [97] had also extended their investigation to study the role of helicity and hydrophobicity in *α*-helical AMP by showing that d-AA substitution at the non-polar face led to stronger antimicrobial activity and lower toxicity owing to the stronger preference toward bacterial cells. Particularly, it was found that high helicity led to stronger antimicrobial activity whereas disruption of the helicity led to decrease in the hydrophobicity and lower hemolytic activity. Both studies revealed that the incorporation of d-AA into the studied peptides affected the helicity and consequently influencing the hydrophobicity and its resulting bioactivity.

Thus far, there have been virtually no studies on the detailed insights into the mechanism of d-AA-containing peptides with other types of structures and targets in spite of the fact that these peptides have already been synthesized and their activities tested, such as in the case of d-AA-containing peptides having *β*-hairpin [48] or *β*-sheet structures [9].

Two available studies had provided a rudimentary look at the potential challenges of understanding the mechanism of d-AA-containing HDPs with structures other than linear [98,99], namely the cyclic polymyxin B (i.e., a natural AMP with a ring structure attached to a largely linear tail). It was found via fluorescence quenching and colorimetry that the ring structure crucially affected both binding to the membrane surface as well as depth of insertion into the membrane. The diastereomer analogues differed from the native all-l form in only the chirality of the ring structure. However, when compared to the all-l peptides the diastereomers binds strongly to the lipid head groups and did not insert deeply into the membrane, which is consistent with the observations for linear, helical peptides. However, instead of preserving antimicrobial activity, the incorporation of d-AAs nearly inactivated the activity of cyclic diastereomer. No explanation was provided, but this result calls into question whether conclusions drawn from d-AA modification of linear peptides can also be extrapolated to the cyclic form.

Another work that provided some insight into the mechanism of non-helical diastereomers was carried out by Sinthuvanich et al. [48], who synthesized an 18-residue diastereomer composed primarily of alternating Val and Lys. The diastereomer exhibited membrane lytic activity against a number of cancer cell lines and had low cytotoxicity against normal mammalian cells such as human umbilical vein endothelial cell (HUVEC). Instrumental analysis and liposomal fluorescence dye leakage assay revealed that the diastereomer was unstructured in solution but adopted a *β*-hairpin structure upon contact with the negatively-charged membrane. The peptide was unstructured in solution because the absence of compensatory interactions and the high density of positively-charged Lys residues resulted in intrastrand charge repulsion and prevented the N- and C-terminal strands from folding into *β*-hairpins. However, once bound to the negatively-charged membranes, the positively-charged Lys were neutralized owing to formation of ion pairs thereby allowing the terminal strands to collapse and form the hairpin structure. Moreover, the alternating placement of Val and Lys residues allows the folded diastereomer to display a polar face comprising of Lys and a hydrophobic face comprising of Val, which consequently conferred the *β*-hairpin structure with the necessary amphipathicity for membrane destabilization. Aside from the study of Sinthuvanich et al. [48], no further noteworthy studies providing insights into the mechanism of non-linear, non-helical diastereomers were found.

### 5.7. Diastereomeric vs. All-*l* and All-*d* Enantiomeric HDPs

Another important point is that the mechanisms of HDPs composed entirely of d-AAs is likely different from that of diastereomers. An all-d enantiomer peptide will not have any difference in peptide property aside from the stereoconfiguration while HDP membrane destabilization is largely independent of the receptor [100] and chirality [15,57,101]. It is therefore likely that all-d enantiomeric HDPs have nearly identical mechanisms of action as their all-l counterparts. Findings from Rodrigues et al. [102] supports this point in which an all-d form of gomesin was found to preserve the anticancer activity of the naturally occuring all-l form. The improvement in lytic activity and the frequently observed apparent increase in the selectivity of all-d enantiomeric HDPs could be explained by their resistance to proteolytic enzymes and possibly by the reduced non-specific protein binding due to stereohindrance thereby allowing greater peptide concentration at the target membrane. However, this statement is far from absolute. Zhan et al. [9] reported an all-d AMP with a selectivity index of over 400. Such a high selectivity index is hard to explain by enzyme resistance and a greater reduction in non-specific binding. The authors argued that a more complicated, stereoconfiguration-sensitive peptide membrane interaction is responsible for such high selective index because several previous works have shown divergent activities including antimicrobial activity between peptide stereoisomers. Whether such a stereochemical effect will occur appears to depend on the sequence and target membrane [103,104]. Baker et al. [105] demonstrated that an all-d-AA magainin derivative was more potent both in vitro and in vivo than their all-l-AA counterpart as they were more cytotoxic towards A549 and could significantly reduce the number and viability of P388D1 lymphoma cells.

## 6. In Vivo Testing of d-AA-Containing HDPs

One of the most important objectives of HDP research is to use them in clinical applications. The development progress of clinically worthy HDPs is slow. As of now, the few instances where HDPs have found mass application in clinical setting were all topical such as gramicidin and polymyxins for primarily treating Gram-positive and Gram-negative bacteria, respectively [106]. Although there are clinically approved peptide-based membrane-disrupting antibiotics available for systemic administration, it is rather questionable whether these antibiotics fit the definition of host defense peptides. An example is daptomycin, which is used as a reserve antibiotic against life-threatening infections such as methicillin-resistant *Staphylococcus aureus* [107]. However, daptomycin is a lipopeptide and works against only Gram-positive bacteria, which is opposite to the equal effectiveness against both Gram-positive and Gram-negative bacteria as displayed by many antimicrobial host defense peptides [7].

Current clinical trials of HDPs is primarily focused on topical applications [108]. Small-scale clinical trials for developing HDPs that are suited for systemic administration are in existence, especially for HDPs conjugated to homing motifs that further enhance their selectivity. Curtis et al. [109] tested a luteinizing-hormone-releasing hormone (LHRH) ligand conjugated to a cationic membrane-disrupting peptide in patients with a range of LHRH receptor-expressing tumor types. However, the results of this study could be described as cautiously optimistic.

In another work, a Phase I clinical trial was initiated with an N-terminal peptide of human lactoferrin for the treatment of infection and inflammation complications resulting from the hematopoietic stem cell transplantation treatment of hematological malignancies [110]. The same peptide initially tested by van der Velden et al. [110] was very well tolerated and is now entering Phase II clinical trials [111]. An essential step in the long journey of a drug from laboratory to clinic is in vivo animal testings. To this end, d-AA incorporation as a means for developing HDPs suited for eventual clinical application deserves special attention because a considerable number of solid results were obtained from in vivo testings of these HDPs.

### 6.1. Targeting Cancer Cells

Papo et al. [13] performed an experiment demonstrating the high in vivo effectiveness of diastereomer HDPs. Metastatic cancer-bearing mice treated with a diastereomer consisting entirely of Leu and Lys survived for no less than 165 days, after which the experiment was terminated. For the entire duration of the test, no change in the HDP dosage was required to maintain negligible tumor growth and strikingly there was zero mortality in the treated animals for the entire duration of the experiment, which is in strike contrast to the 100% mortality after 75 days in the control group. Furthermore, there were no noteworthy side effects of the peptide, and blood tests showed that the peptide did not adversely affect the healthy control mice either. The diastereomer peptide was shown to act via membrane disruption although partial apoptotic activity could not be ruled out.

The above results are significant for several reasons as follows:

1. The benefit of long-term survival with nearly no side effects was demonstrated.
2. The tumor was unable to develop resistance against the diastereomeric peptide tested by Papo et al. [13] even after prolonged exposure. This finding is in line with a previous observation by Hilchie et al. [2], who reported that no acquisition of resistance to HDPs by cancer has ever been documented. Furthermore, l-AA AMPs have been shown to induce resistance in bacteria when applied using graded doses for a prolonged period of time [8]. This finding may be due to the greater mutation potential of prokaryotes than that of eukaryotic cancer cells. However, a gradient exposure study performed with an all-d AMP in *E. coli* and *S. aureus* showed that resistance did not develop against the all-d AMP [9]. This result indicated that the inclusion of d-AAs not only stabilizes the peptide against serum proteases but also likely protects them against bacterial proteases. Thus, d-AA inclusion as a method of boosting HDP resistance against pathogen evasion is an avenue that cannot be overlooked.
3. The diastereomeric peptide tested by Papo et al. [13] was suitable for systemic application and was not conjugated to any homing motifs, nor did it depend on any delivery vesicles. The development of anticancer HDPs suited for systemic administration is important because this application remains the only route for combating metastatic cancer.

Previous systemic administration of membrane lytic peptides almost invariably required a homing domain. Mostly, for in vivo experiments, anticancer HDPs were not administered systemically but instead the experiments used local or intratumoral injection and most significantly nearly all previous tests had little to no influence on metastatic cancer [13]. Therefore, this study provided a proof of concept that diastereomeric anticancer HDPs can be very effective as long-term metastatic cancer management agents. The results demonstrated in a practical setting that these diastereomeric HDPs are able to overcome many of the issues faced by HDPs in clinical applications such as short serum life [10], sensitivity to proteases [47], often inadequate selectivity and potentially damaging antigenicity [11,112].

A similar achievement was presented by Papo et al. [113] where two diastereomers composed solely of Leu and Lys (i.e., similar to the diastereomer also tested by Papo et al. [13]) were tested for their capacity to inhibit metastatic melanoma in mice. One of the two tested peptides exhibited highly selective toxicity towards cancer cells with minimal side effects when systemically administered. No weakness or loss of weight was observed throughout the entire experiment and postmortem histopathological evaluation revealed that despite the intravenous injection, the diastereomer did not cause damage to any organs.

Makovitzki et al. [114] tested three diastereomers in human prostate carcinoma xenograft-bearing mice. The tested diastereomers were rich in histidine, thus making them sensitive to environmental pH. Because solid tumors have high metabolic rates and poor vasculature, their local environment tends to be anaerobic and acidic because ATP hydrolysis results in the production of lactic acid and because of the poor washout of the acidic products [115]. A drug that can react to environmental pH would have the advantage of selectivity by becoming active in only abnormal pH conditions. Because the tested diastereomers of Makovitzki et al. [114] possessed a significant percentage of histidine residues, they tended to become positively-charged only in acidic environments; thus, the peptides were more prone to bind to membranes only in the vicinity of tumors and had a decreased probability of non-specific toxicity. The peptides were administered both intratumorally and systemically. For both routes, the peptides were effective at reducing both the tumor weight and the tumor blood vessel growth. One of the three tested diastereomers exhibited acute systemic toxicity at a concentration above 8 mg/kg. However, the other two had no observable toxicity at concentrations of up to 30 and 20 mg/kg. Ellerby et al. [116] also demonstrated a strong systemic anti-metastatic cancer effect in which an all-d enantiomer AMP was conjugated to tumor blood vessel homing motifs consisting either Cys-Asn-Gly-Arg-Cys (CNGRC) or doubly cyclized RGD (RGD-4C). The peptide displayed no lytic activity against mammalian cells but induced apoptosis by disrupting the negatively-charged mitochondrial membrane once internalized into the cytoplasm. The peptide conjugate significantly prolonged the survival of metastatic cancer-bearing mice.

In addition to systemic administration, d-AA-containing HDPs have been tested for treating cancer in model animals by other administration routes. Although these processes did not provide coverage as thorough as for systemic administration, impressive results were nevertheless achieved and included multiple instances of complete tumor ablation, which essentially cured the animal of cancer. Additionally, peptides used in these studies were not administered systemically nor were there evidence that the studied peptides cannot be systemically administered or further modified to make them suitable for systemic use. Baker et al. [105] tested a series of magainin analogues for their inhibitory activity against ovarian cancer when injected intraperitoneally. One of the analogues, an all-d enantiomer, proved to be as effective as doxorubicin at inhibiting tumor growth and was the most active of the analogues tested. Some side effects were observed in the form of local irritation, resulting in adhesions between the serosal surfaces of the abdominal organs; however the study did not discuss the severity of the side effects as compared to those of doxorubicin. It is unlikely that the HDP side effects were comparable to those of doxorubicin. Papo et al. [44] tested a diastereomer on a prostate cancer xenograft-bearing mice. The diastereomer was able to completely arrest tumor growth when administered intratumorally, whereas its l-AA parent peptide was able to only slow tumor growth. In fact, the diastereomer was so effective that the tumor completely disappeared in 40% of the test animals. Figure S6 shows the atrophied tumor at the end of the test. Chen et al. [95] conjugated the integrin homing domain (RGD) to the AMP tachyplesin. The construct was able to induce tumor apoptosis via disruption of the negatively-charged mitochondrial membrane when injected intraperitoneally into a melanoma xenograft-bearing mice.

### 6.2. Targeting Bacterial Cells

Compared to cancer treatment, diastereomer HDPs have had a longer history of usage in fighting bacterial infections, which included actual clinical applications such as the natural diastereomer gramicidin as discussed earlier. This section focuses on non-topical administration routes because the topical application of AMPs is well documented.

Braunstein et al. [46] compared a diastereomer AMP to its l-AA parent, which were both consisted of only Leu and Lys, for the treatment of mice infected with gentamicin-sensitive *P. aeruginosa* and gentamicin-resistant *A. baumannii* bacteria. To reduce the influence of the immune system, the mice were rendered transiently neutropenic by cyclophosphamide treatment. The pathogen-inoculated mice became weak and less active after just one day and death occurred between 2 to 4 days. The diastereomer but not its l-AA parent peptide cured the infection in nearly 80% of instances and was equally effective against both gentamicin-sensitive *P. aeruginosa* and gentamicin-resistant *A. baumannii* bacteria. Although gentamicin was slightly more effective than the diastereomer against the gentamicin-sensitive *P. aeruginosa*, it was completely useless against the gentamicin-resistant *A. baumannii*. Physical improvement after diastereomer administration was observed between 3 to 5 days after the bacterial challenge. Mortality was monitored for at least 10 days after treatment and all survivors recovered their physical activity. Both the diastereomer and all-l enantiomer peptides were tested for acute toxicity, and mortality occurred when the peptides were injected intravenously at 6 mg/kg dissolved in 0.25 mL buffer. Interestingly, when dissolved in 0.9 mL of buffer, no mortality occurred despite the use of the same 6 mg/kg dose of peptide. Blood chemistry tests revealed no abnormality when the peptide was used in safe doses.

Wang et al. [1] combined peptide library screening with structure-based approach to design a family of diastereomers targeted to a number of commonly faced pathogenic bacteria named ESKAPE pathogens. One of the designer diastereomers termed 17BIPHE2 exhibited activity against all ESKAPE pathogens in vitro and could prevent staphylococcal biofilm formation in a catheter-associated infection mouse model. A hemolytic assay showed that the majority of the diastereomers had 50% hemolysis (HL50) at a concentration greater than 500 μM, whereas the most potent 17BIPHE2 showed an HL50 of 225 μM; in contrast, the mean MIC value was just 3.1 μM against the ESKAPE pathogens.

Lan et al. [117] tested 6 analogous all-d enantiomers against the extensively drug-resistant *Mycobacterium tuberculosis* (XDR-TB). The study used human monocytic cells THP-1 as macrophage models in both in vitro and ex vivo settings and the results demonstrated the effectiveness of all six peptides at neutralizing the XDR-TB bacterial colony in vitro. Importantly, the peptides were effective at breaking down the heavily aggregated XDR-TB colonies. The author postulated but did not prove that the breakdown of the bacterial aggregates was due to the amphipathic peptides acting as detergents and reducing the hydrophobic interactions between the highly lipidic cell walls of the mycobacteria. The peptides were unable to eradicate intracellular mycobacteria, although the intracellular growth of the bacteria was substantially inhibited.

### 6.3. Targeting Other Pathogens

There are only a limited number of in vivo tests that have been carried out for evaluating the activity of d-AA-containing HDPs against diseases other than bacterial infection and cancer. One interesting study, despite being ex vivo rather than in vivo was carried out by Lynn et al. [4], which demonstrated the capacity of the d-enantiomer of BMAP-28 (i.e., a cathelicidin analogue) in neutralizing the protozoan *Leishmania* inside living macrophages. *Leishmania* possesses two forms, the free living promastigote form and the parasitic intracellular amastigote form. *Leishmania* is typically treated with polyvalent antimony. However, increased incidence of antimony resistance by *Leishmania* has been observed [118]. Previous works have demonstrated the effectiveness of various HDPs at neutralizing *Leishmania* promastigotes via the characteristic HDP membrane depolarization; however, there were no reports of activity against the intracellular amastigote form. The study by Lynn et al. [4] demonstrated that both l- and d-enantiomers of BMAP-28 were able to greatly reduce the *Leishmania* amastigotes burden inside mouse macrophages in which the d-enantiomer was twice as effective as the l-enantiomer. In vitro tests showed that both the d and l forms of BMAP-28 could neutralize *Leishmania* promastigotes via membrane depolarization and late-stage apoptosis. However, this study did not explain how the peptide works against *Leishmania* amastigotes and how the peptide enters the infected macrophage.

In addition to treating infections in animals, HDPs have been found to defend plants both locally and systemically from bacterial and fungal infection. Brotman et al. [119] synthesized cationic diastereomeric peptides consisting of only four residues conjugated to fatty acids. According to the study, this type of lipopeptide is considered a subfamily of HDPs. These lipopeptides can act via membrane lysis and can protect against bacterial and fungal infection when applied directly to the infected part of the plant while not harming the plant tissue [120]. Additionally, the peptides could prime the systemic induction of antimicrobial compounds. In the study by Brotman et al. [119], *Arabidopsis* plant seedlings and cucumber leaves were rendered resistant to *P. syringae* bacteria and *B. cinerea* fungi if suspended in the peptide solution 24 h before bacterial challenge. The peptide afforded no protection if both the bacteria and peptide were suspended together with the seedling, indicating that the peptide worked more like a vaccine. A gene expression assay revealed the activation of defense-related genes and the systemic induction of antimicrobial compounds. The defensive effects induced by peptide treatment lasted at least 4 days. López-García et al. [121] synthesized a series of all-d enantiomeric hexapeptides through the screening of a synthetic peptide combinatorial library in a positional scanning format. The peptides were tested in vivo for their capacity to delay the decay of orange fruits via a wound infection assay. One of the peptides could keep the wound free of infection in 38% of the cases for 3 days.

## 7. Toxicity

One important aspect of d-AA-containing HDPs that is under addressed in the current literature is the toxicity of this class of HDPs against healthy mammalian cells. Low toxicity against healthy mammalian cells is vital for the clinical applicability of a peptide. However, the toxicity of virtually all tested d-AA-containing HDPs against healthy mammalian cells was simply inferred from its IC_50_ value with no discussion on how and why off-target toxicity occurs. To the best of our knowledge, no dedicated mechanism study exists on the toxicity of d-AA-containing HDPs against healthy mammalian cells. Although the toxicity against healthy mammalian cells by d-AA-containing HDPs (i.e., whether as diastereomer or all-d enantiomer) were generally lower than their all-l counterparts. However, it should be noted that toxicity was not absent as toxicity toward healthy control cells was on par or even higher than the toxicity against target cells [45,66,122], thus highlighting the need for further study in this area. It should also be noted that the reported bioactivity of d-AA-containing HDPs together with their all-l counterparts were not often found as d-AA-containing HDPs were typically tested alone. Another major issue in the exploration of off-target toxicity of d-AA-containing HDPs is the limited spectrum of healthy mammalian control cells tested by many studies. Often, only a few control cell lines were used. Considering the importance of low off-target toxicity and the absence of dedicated mechanism study, further research into how and why toxicity against healthy mammalian cells by d-AA-containing HDPs occurs could be of vital importance in facilitating further clinical applications. A recent study from Reay et al. [123] addressed this nascent and important area of research on d-AA-containing HDPs by investigating the in vivo toxicity of the d-isoform of the 8K-wild-type-NBD peptide (i.e. where NBD is an acronym for NEMO-binding domain and NEMO is an acronym for NF-*κ*B essential modulator). At the onset, Reay et al. [123] hypothesized that the d-isoform may potentially have higher therapeutic effect than their l-isoform owing to their longer persistence in vivo due to their inherent protease resistance. Their findings indicated that although the d-isoform afforded a similar level of bioactivity as the l-isoform but to their surprise the renal toxicity of the d-isoform was significantly higher and this was attributed to inherent toxicity of d-Ser as described previously by Krug et al. [124]. However, it should be noted that the d-isoform of the 8K-wild-type-NBD peptide is an all-d peptide comprising of a staggering number of nineteen d-AA (i.e., eight d-Lys and eleven d-AA on the NBD). Such a high number of d-AA on the HDP has been suggested by Huang et al. [96] to be unfavorable for the bioactivity. Particularly, their study observed that when the number of d-AA in the peptide was more than three, deterioration of the anticancer activity was observed.

## 8. Synthesis of d-AA-Containing HDPs

d-AA-containing HDPs could be produced by chemical synthesis or enzymatic synthesis [12]. In the former approach, d-AA can be produced via chiral resolution of l-amino acids or by asymmetric synthesis from chiral or prochiral starting materials. The inherent racemization of d-AA leads to low yields and high cost thereby making chemical synthesis unfavorable. Enzymatic synthesis have thus been proposed to be a promising approach for producing d-AA with high optical purity as well as being cost-efficient and environmental friendly. Briefly, enzymatic synthesis of d-AA can be carried out using three major classes of enzymes namely hydrolases, oxidoreductases and d-amino acid aminotransferases [12].

Several enzymes are involved in the synthesis of d-AA via the hydrolase enzymes consisting of d-hydantoinase coupled with *N*-carbamoyl-d-amino acid amidohydrolase, *N*-acyl-d-amino acid amidohydrolase, d-amino acid amidase and d-amino peptidase. Firstly, a two-step catalysis of the starting material dl-5-substituted hydantoins via d-hydantoinase coupled with *N*-carbamoyl-d-amino acid amidohydrolase have been successfully applied for d-AA production at the industrial scale [125]. This methods have been used to produce a wide range of d-AA including d-phenylglycine, d-*p*-hydroxyphenylglycine, d-Trp, d-Phe, d-Val, d-Ala and d-Met [126]. Secondly, hydrolysis of *N*-acyl-d-amino acid via *N*-acyl-d-amino acid amidohydrolase is the most useful and convenient reaction for producing d-AA. Products obtained from this method includes fatty acid and corresponding d-AAs comprising of d-Ala, d-Arg, d-Asp, d-Glu and d-Leu [17]. Thirdly, d-amino acid amidase can catalyze the stereospecific hydrolysis of d-AA amide to obtain ammonia and the corresponding d-AAs. This method have been successfully applied in the synthesis of d-phenylalaninamide, d-tyrosinamide, d-leucinamide, d-methioninamide, d-glutaminamide and d-lysinamide [127,128]. Moreover, other hydrolases targeting peptide substrates were used to produce d-AAs. For example, d-aminopeptidase preferred to catalyze peptide substrates for producing d-Ala with 100% yield and >99% enantiomeric excess [129]. Alkaline d-peptidase catalyze peptides that are composed of aromatic d-AAs via hydrolysis reaction of the carboxy-terminal peptide of (d-Phe)_3_ and (d-Phe)_4_ to produce d-Phe and (d-Phe)_2_ [130].

l-amino acid oxidase and d-amino acid dehydrogenase are two important enzymes belonging to the oxidoreductase class. Firstly, l-amino acid oxidase catalyzes the conversion of l-amino acids with strict stereospecificity to produce d-AA via deamination reaction with a maximum of 50% theoretical yield. Comparing with d-hydantoinases and d-aminoacylases, this method gain beneficial of producing d-Glu, d-Arg and d-homoserine. Second, d-amino acid dehydrogenase have been used to produce d-AAs in the presence of NADPH. Synthesis of d-AA against a series of *α*-keto acids produces corresponding d-AAs through amination reaction. However, only *meso*-diaminopilemate dehydrogenase from *Symbiobacterium thermophilum* IAM14863 is able to synthesize d-AA while *meso*-diaminopilemate dehydrogenase from other organisms do not afford the activity to produce d-AA, which limits the potential application of these enzymes for large scale production of d-AAs [12].

d-amino acid aminotransferase is the last class of enzymes involved in d-AA synthesis. The principle of this class relies on the transfer of amino group from d-AA donors to keto-acid acceptors. Many types of keto-acids are used as starting materials and can be utilized to produce d-AAs at different rate of activity by d-amino acid aminotransferase from *Lactobacillus salivarius* [131]. Moreover, the use of a thermostable d-amino acid aminotransferase from *Bacillus* sp. coupled with glutamate racemase, glutamate dehydrogenase and formate dehydrogenasen as a multienzyme system have been employed for catalyzing the synthesis of d-Phe and d-Tyr using phenylpyruvate and hydroxyphenylpyruvate as starting materials [132].

In regards to the identification of bioactive d-AA peptides, mirror-inspired approaches such as mirror-image phage display and retro-inverso approach have been suggested as promising methods. Briefly, the mirror-image phage display approach originally proposed by Schumacher et al. [133] encompasses the chemical synthesis of a d-enantiomer of the protein, using this d-enantiomer to identify interacting l-peptide ligands via phage display screening, synthesize a d-enantiomer of the aforementioned l-peptide and finally this d-enantiomer should be able to interact with the target l-enantiomeric protein. Goodman and Chorev [134] proposed a simpler approach in which retro-inverso peptides are comprised of d-AA in the reverse sequence order to that of the natural l-AA peptide.

## 9. Computational Modeling of d-AA-Containing HDPs

As structure governs the activity of peptides, therefore the understanding of such structure-activity relationship via quantitative structure-activity relationship (QSAR) is crucial towards designing robust therapeutic peptides [135,136]. In QSAR studies of peptides, *z*-scale descriptors [137] are widely used but are unable to describe the d-AAs. Furthermore, more comprehensive softwares for computing molecular descriptors such as PROFEAT [138] and protr [139] also undergo the same problem as they too do not differentiate between the l and d isoform, which therefore poses a problem for studies investigating d-AA-containing peptides. A possible solution to this may be to utilize computational chemistry for ab initio geometry optimization of the structure followed by calculation of the descriptors. However, the disadvantage of this approach is that it would be rather time-consuming, which is particularly true if the employed theoretical level is computationally demanding such as density functional theory or Møller-Plesset perturbation theory.

In spite of some of the aforementioned hurdles, there is a growing momentum of recent studies that are implementing support (i.e., development of forcefields, custom configuration, etc.) for non-canonical amino acids such as d-AAs. For example, PEPstrMOD [140] allows structure prediction of peptides encompassing the set of 20 canonical amino acids as well as the 210 non-canonical amino acids as described in SwissSideChain [141]. Yongye et al. [142] employed molecular dynamics simulation to investigate the cyclization of d-AA-containing peptides. Yet another study [143], devised an approach known as PEPOP that allows the prediction of the immunogenicity of peptides containing both l-AA and d-AA. Lastly, in regards to the aforementioned point on computing molecular descriptors of d-AAs, a possible solution that may not have been performed before, would be to use circular fingerprints [144,145,146] to describe the d-AAs in which the atomic features of the amino acid is considered in a radial fashion.

## 10. Conclusions

Putting everything into perspective, it is clear that d-AA substitution, whether partial or complete, is a highly effective way to improve pharmacokinetic properties and therapeutic effectiveness. This process offers a relatively easy way to overcome a number of hurdles faced by HDPs. This review provides the first dedicated systematic look at the influence of d-AAs on HDP properties and mechanisms as well as the state of their utilization in the development of novel HDPs. To date, a considerable number of d-AA-containing HDPs have been synthesized and tested for activity. However, only a fraction of the tested peptides had approached clinical requirements. More importantly is the fact that the mechanism by which they function is rather poorly understood, particularly how their mechanism differs from their l-AA counterparts. Although many research works had attempted to elucidate the mechanisms of the studied peptides, however such studies tended to be rudimentary and proved what is already known while dedicated mechanism studies are few and old. There is little doubt that further exploration of d-AA substitution at both the theoretical and practical level will be a fertile scientific field that could accelerate the clinical application of HDPs.

## Supplementary Materials

Supplementary materials can be found at http://www.mdpi.com/1422-0067/17/7/1023/s1.
