# Supplementary material for: Roles of d-Amino Acids on the Bioactivity of Host Defense Peptides
Source: Int J Mol Sci. 2016 Jun 30;17(7):1023. doi: 10.3390/ijms17071023 (PMC4964399; doi:10.3390/ijms17071023)
Supplement: Supplementary file 1 [file ijms-17-01023-s001.zip › ijms-134588-Supplementary Materials/Rightslink Printable License (Figure S2 and S3).pdf]

Copyright Clearance Center RightsLink®

Home | Account Info | Help | Live Chat

**Title:**	A Novel Lytic Peptide Composed of dl-Amino Acids Selectively Kills Cancer Cells in Culture and in Mice
**Author:**	Niv Papo, Michal Shahar, Lea Eisenbach, Yechiel Shai
**Publication:**	Journal of Biological Chemistry
**Publisher:**	The American Society for Biochemistry and Molecular Biology
**Date:**	Jun 6, 2003

Copyright © 2003, by the American Society for Biochemistry and Molecular Biology

Logged in as:
Chanin Nantasenamat
Account #:
3000896297

LOGOUT

Non-profit/Non-commercial Use

For **Non-profit/Non-commercial** uses: You are free to copy, distribute, transmit and to adapt the work under the following conditions:

Attribution. You must attribute the work in the manner specified by the author or licensor (but not in any way that suggests that they endorse you or your use of the work).

Non-commercial. You may not use the work for commercial purposes; including original authors reusing content by a commercial publisher.

BACK | CLOSE WINDOW

Copyright © 2015 Copyright Clearance Center, Inc. All Rights Reserved. Privacy statement. Terms and Conditions.
Comments? We would like to hear from you. E-mail us at customercare@copyright.com
