# Supplementary material for: Roles of d-Amino Acids on the Bioactivity of Host Defense Peptides
Source: Int J Mol Sci. 2016 Jun 30;17(7):1023. doi: 10.3390/ijms17071023 (PMC4964399; doi:10.3390/ijms17071023)
Supplement: Supplementary file 1 [file ijms-17-01023-s001.zip › ijms-134588-Supplementary Materials/Rightslink Printable License (Figure S5).pdf]

AMERICAN ASSOCIATION FOR CANCER RESEARCH LICENSE TERMS AND CONDITIONS

Jul 15, 2015

This is a License Agreement between Chanin Nantasenamat ("You") and American Association for Cancer Research ("American Association for Cancer Research") provided by Copyright Clearance Center ("CCC"). The license consists of your order details, the terms and conditions provided by American Association for Cancer Research, and the payment terms and conditions.

License Number	3670570102234
License date	Jul 15, 2015
Licensed content publisher	American Association for Cancer Research
Licensed content publication	Cancer Research
Licensed content title	RGD-Tachyplesin Inhibits Tumor Growth
Licensed content author	Yixin Chen, Xueming Xu, Shuigen Hong et al.
Licensed content date	March 3, 2001
Volume number	61
Issue number	6
Type of Use	Journal/Magazine
Requestor type	academic/educational
The new work is a scholarly article, a review article, or content to be included in a chapter of a book	yes
Format	print and electronic
Portion	figures/tables/illustrations
Number of figures/tables/illustrations	1
Will you be translating?	no
Circulation	100000
Territory of distribution	Worldwide
Order reference number	Figure9
Title of the article	Roles of D-Amino Acids on the Bioactivity of Host Defense Peptides
Publication the new article is in	Peptides
Publisher of the article	Elsevier
Author of new article	Hao Li, Virapong Prachayasittikul, Chanin Nantasenamat
Expected publication date	Dec 2015
Estimated size of the article (pages)	9
Billing Type	Credit Card

Credit card info	Master Card ending in 2117
Credit card expiration	06/2019
Total	940.50 USD

Terms and Conditions

American Association for Cancer Research (AACR) Terms and Conditions

INTRODUCTION

The Publisher for this copyright material is the American Association for Cancer Research (AACR). By clicking "accept" in connection with completing this licensing transaction, you agree to the following terms and conditions applying to this transaction. You also agree to the Billing and Payment terms and conditions established by Copyright Clearance Center (CCC) at the time you opened your Rightslink account.

LIMITED LICENSE

The AACR grants exclusively to you, the User, for onetime, non-exclusive use of this material for the purpose stated in your request and used only with a maximum distribution equal to the number you identified in the permission process. Any form of republication must be completed within one year although copies made before then may be distributed thereafter and any electronic posting is limited to a period of one year. Reproduction of this material is confined to the purpose and/or media for which permission is granted. Altering or modifying this material is not permitted. However, figures and illustrations may be minimally altered or modified to serve the new work.

GEOGRAPHIC SCOPE

Licenses may be exercised as noted in the permission process

RESERVATION OF RIGHTS

The AACR reserves all rights not specifically granted in the combination of 1) the license details provided by you and accepted in the course of this licensing transaction, 2) these terms and conditions , and 3) CCC's Billing and Payment terms and conditions.

DISCLAIMER

You may obtain permission via Rightslink to use material owned by AACR. When you are requesting permission to reuse a portion for an AACR publication, it is your responsibility to examine each portion of content as published to determine whether a credit to, or copyright notice of a third party owner is published next to the item. You must obtain permission from the third party to use any material which has been reprinted with permission from the said third party. If you have not obtained permission from the third party, AACR disclaims any responsibility for the use you make of items owned by them.

LICENSE CONTINGENT ON PAYMENT

While you may exercise the rights licensed immediately upon issuance of the license at the end of the licensing process for the transaction, provided that you have disclosed complete and accurate details of your proposed use, no license is finally effective unless and until full payment is received from you, either by the publisher or by the CCC, as provided in CCC's Billing and Payment terms and conditions. If full payment is not received on a timely basis, then any license preliminarily granted shall be deemed automatically revoked and shall be void as if never granted. Further, in the event that you breach any of these terms and conditions, or any of the CCC's Billing and Payment terms and conditions, the license is automatically revoked and shall be void as if never granted. Use of materials as described in a revoked license, as well as any use of the materials beyond the scope of an unrevoked license, may constitute copyright infringement and the publisher reserves the right to take

any and all action to protect its copyright in the materials.

COPYRIGHT NOTICE
You must include the following credit line in connection with your reproduction of the licensed material: "Reprinted (or adapted) from Publication Title, Copyright Year, Volume/Issue, Page Range, Author, Title of Article, with permission from AACR".

TRANSLATION
This permission is granted for non-exclusive world English rights only.

WARRANTIES
Publisher makes no representations or warranties with respect to the licensed material.

INDEMNIFICATION
You hereby indemnify and agree to hold harmless the publisher and CCC, and their respective officers, directors, employees and agents, from and against any and all claims arising out of your use of the licensed material other than as specifically authorized pursuant to this license.

REVOCATION
The AACR reserves the right to revoke a license for any reason, including but not limited to advertising and promotional uses of AACR content, third party usage and incorrect figure source attribution.

NO TRANSFER OF LICENSE
This license is personal to you and may not be sublicensed, assigned, or transferred by you to any other person without publisher's written permission.

NO AMENDMENT EXCEPT IN WRITING
This license may not be amended except in a writing signed by both parties (or, in the case of publisher, by CCC on publisher's behalf).

OBJECTION TO CONTRARY TERMS
Publishers hereby objects to any terms contained in any purchase order, acknowledgement, check endorsement or other writing prepared by you, which terms are inconsistent with these terms and conditions or CCC's Billing and Payment terms and conditions. These terms and conditions together with CCC's Billing and Payment terms and conditions (which are incorporated herein) comprise the entire agreement between you and publisher (and CCC) concerning this licensing transaction. In the event of any conflict between your obligations established by these terms and conditions, and those established by CCC's Billing and Payment terms and conditions, these terms and conditions shall control.

THESIS/DISSERTATION TERMS
If your request is to reuse an article authored by you and published by the AACR in your dissertation/thesis, your thesis may be submitted to your institution in either in print or electronic form. Should your thesis be published commercially, please reapply.

ELECTRONIC RESERVE
If this license is made in connection with a course, and the Licensed Material or any portion thereof is to be posted to a website, the website is to be password protected and made available only to the students registered for the relevant course. The permission is granted for the duration of the course. All content posted to the website must maintain the copyright information notice.

JURISDICTION

This license transaction shall be governed by and construed in accordance with the laws of Pennsylvania. You hereby agree to submit to the jurisdiction of the federal and state courts located in Pennsylvania for purposes of resolving any disputes that may arise in connection with this licensing transaction.

Other Terms and Conditions: None

v1.0

Questions? customercare@copyright.com or +1-855-239-3415 (toll free in the US) or +1-978-646-2777.
