# Supplementary figures and images for: Roles of d-Amino Acids on the Bioactivity of Host Defense Peptides
Source: Int J Mol Sci. 2016 Jun 30;17(7):1023. doi: 10.3390/ijms17071023 (PMC4964399; doi:10.3390/ijms17071023)

**A**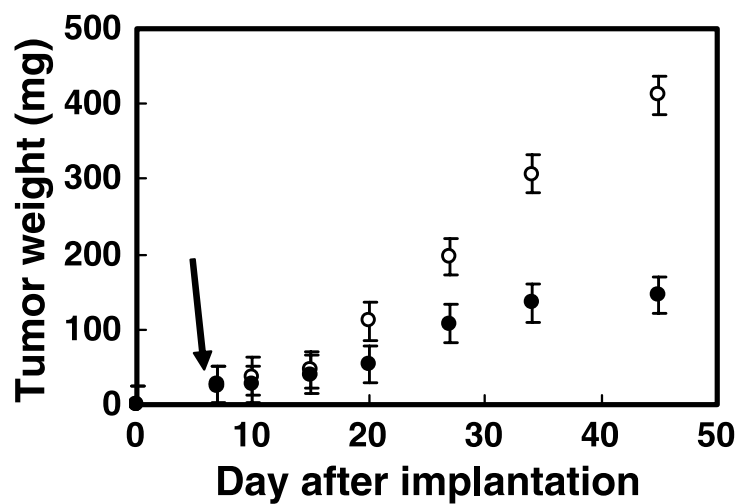**B**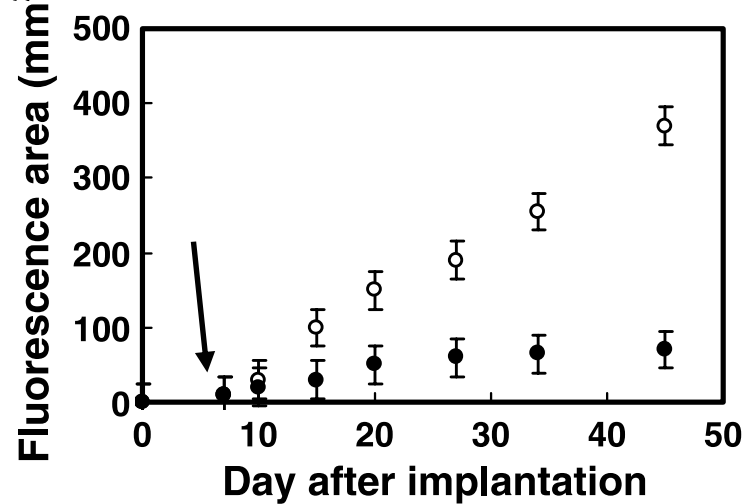**C**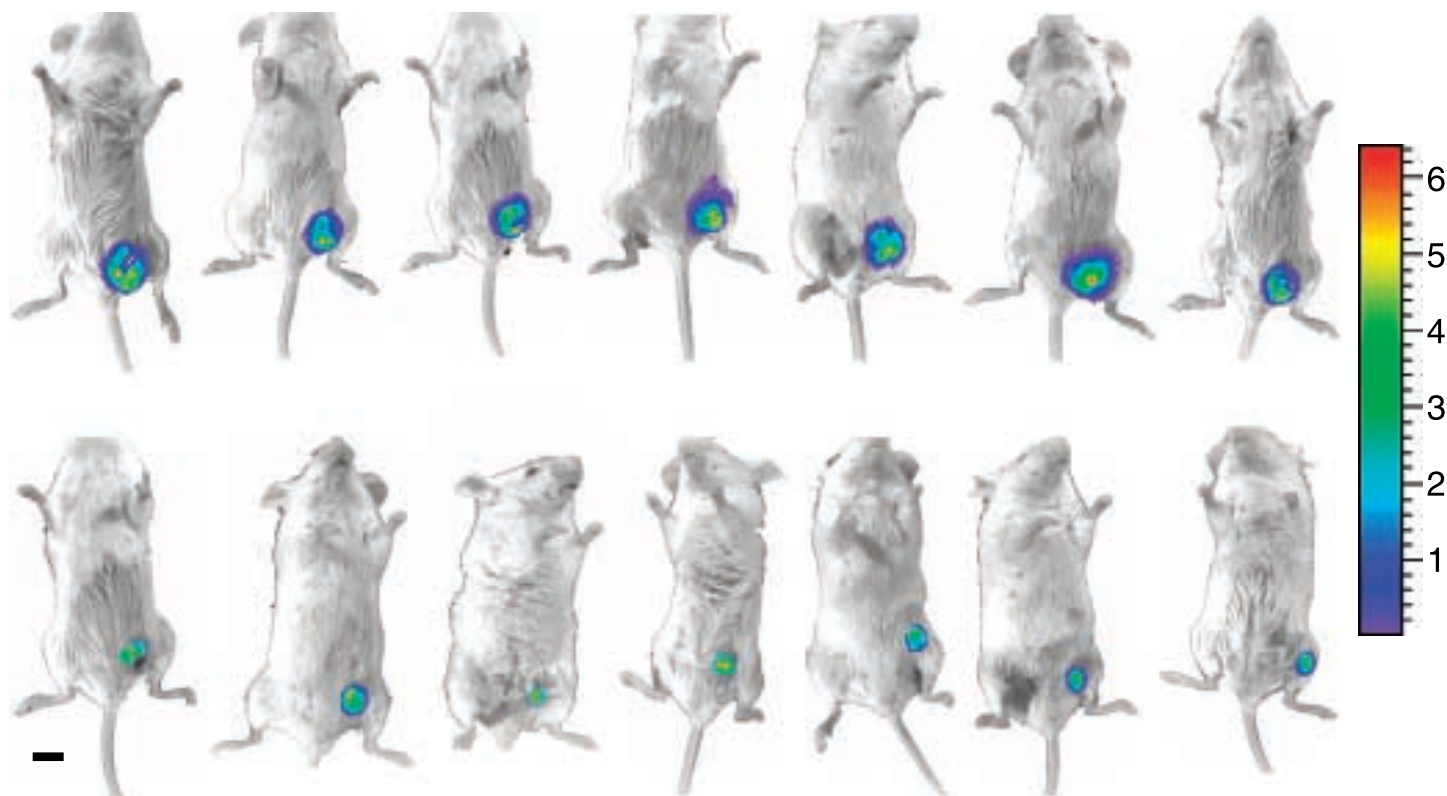**D**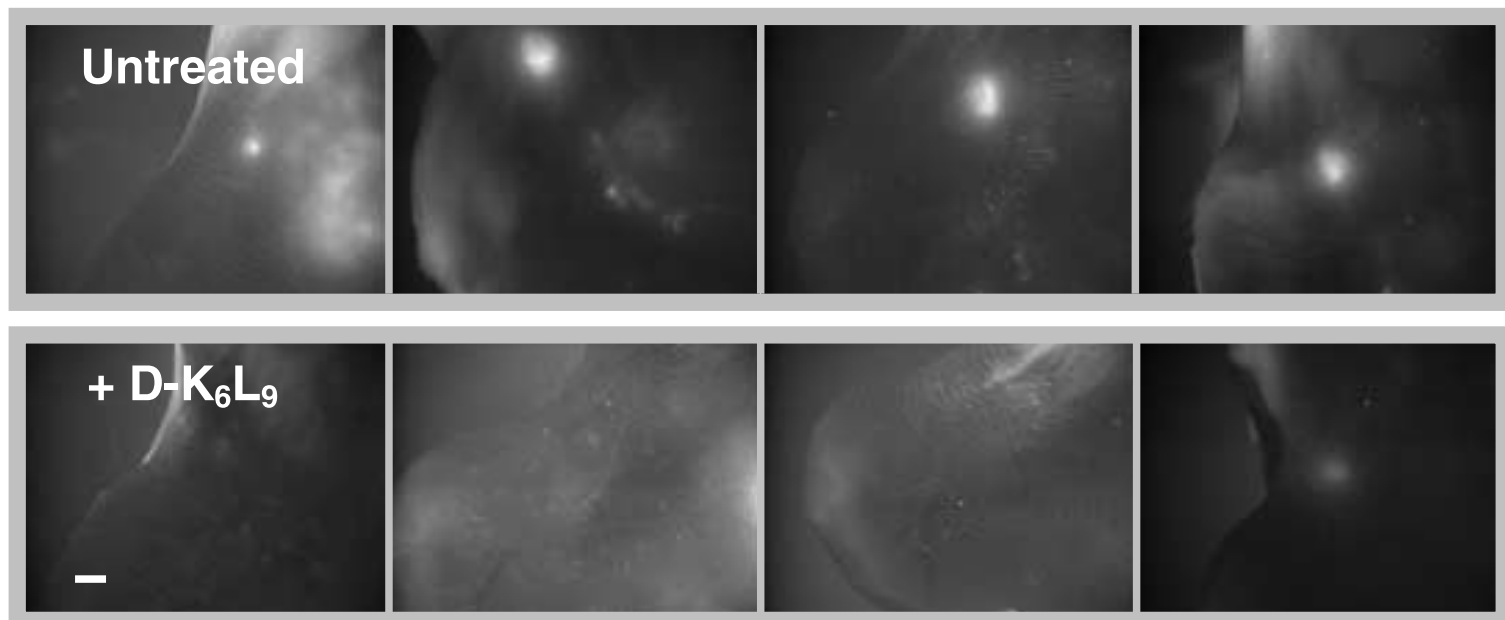

Supplement: Supplementary file 1 [file ijms-17-01023-s001.zip › ijms-134588-Supplementary Materials/HDP_Supp_Figure1.pdf]

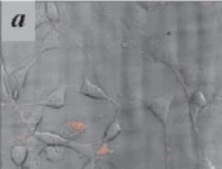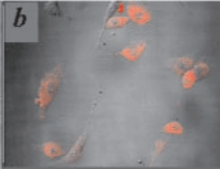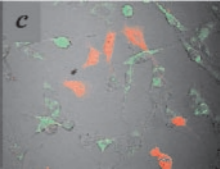

Supplement: Supplementary file 1 [file ijms-17-01023-s001.zip › ijms-134588-Supplementary Materials/HDP_Supp_Figure2.pdf]

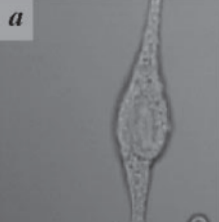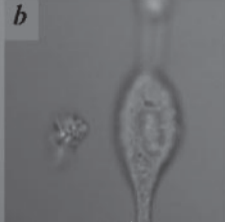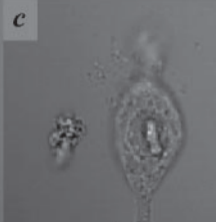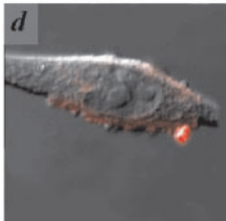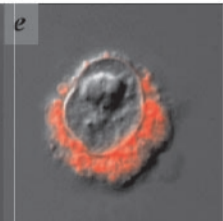

Supplement: Supplementary file 1 [file ijms-17-01023-s001.zip › ijms-134588-Supplementary Materials/HDP_Supp_Figure3.pdf]

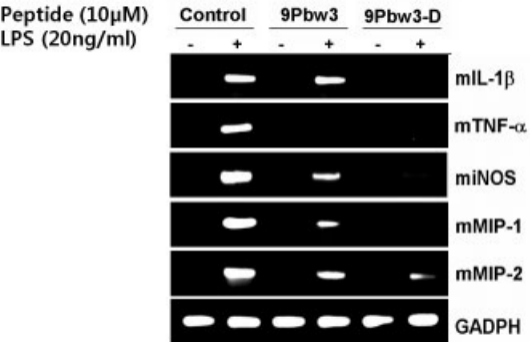

Supplement: Supplementary file 1 [file ijms-17-01023-s001.zip › ijms-134588-Supplementary Materials/HDP_Supp_Figure4.pdf]

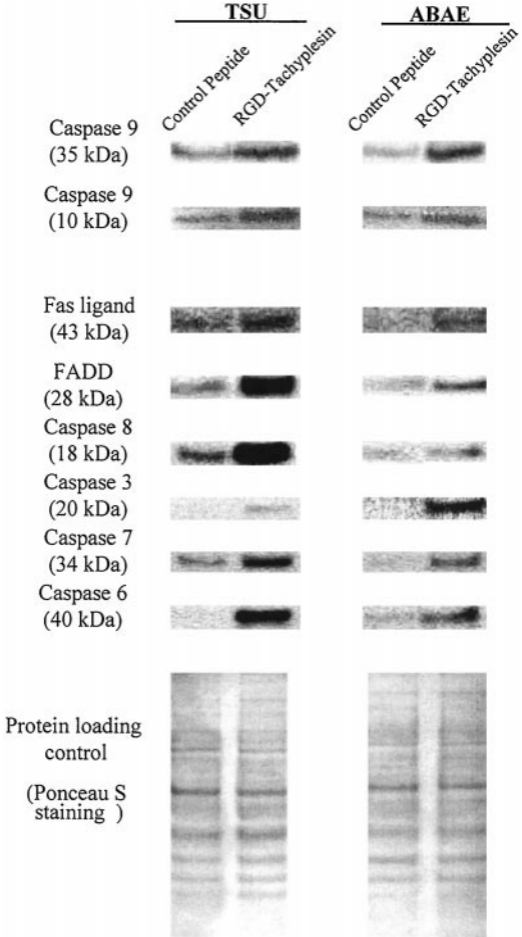

Supplement: Supplementary file 1 [file ijms-17-01023-s001.zip › ijms-134588-Supplementary Materials/HDP_Supp_Figure5.pdf]

**A**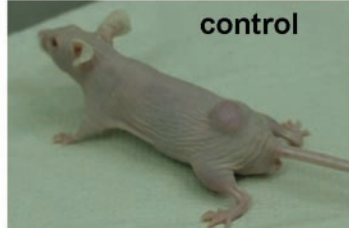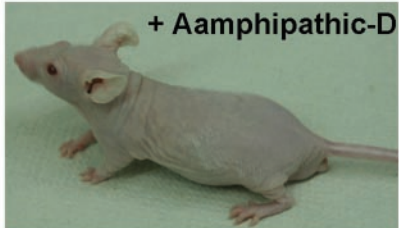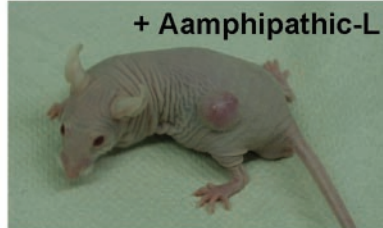**B**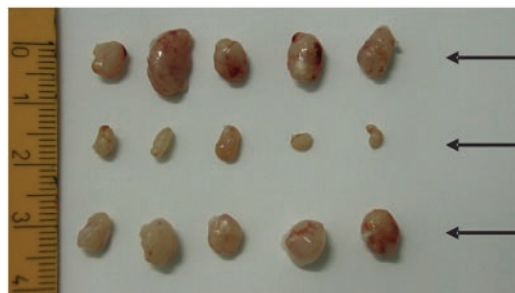

control

+ Aamphipathic-D

+ Aamphipathic-L

**C**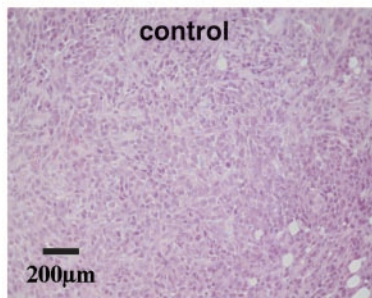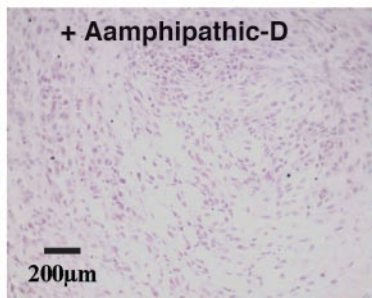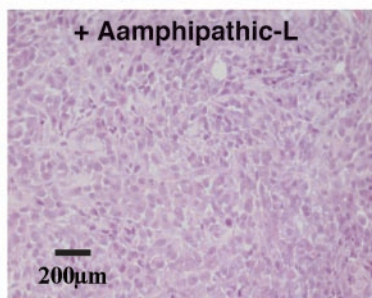

Supplement: Supplementary file 1 [file ijms-17-01023-s001.zip › ijms-134588-Supplementary Materials/HDP_Supp_Figure6.pdf]
